# Transcriptome analysis of *Escherichia coli* K1 after therapy with hesperidin conjugated with silver nanoparticles

**DOI:** 10.1186/s12866-021-02097-2

**Published:** 2021-02-17

**Authors:** Abdulkader Masri, Naveed Ahmed Khan, Muhammad Zarul Hanifah Md Zoqratt, Qasim Ayub, Ayaz Anwar, Komal Rao, Muhammad Raza Shah, Ruqaiyyah Siddiqui

**Affiliations:** 1 Department of Biological Sciences, School of Science and Technology, Sunway University, Bandar Sunway, Malaysia; 2 Department of Clinical Sciences, College of Medicine, University of Sharjah, University City, Sharjah, United Arab Emirates; 3 Monash University Malaysia Genomics Facility, School of Science, 47500 Bandar Sunway, Selangor Darul Ehsan Malaysia; 4 H.E.J. Research Institute of Chemistry, International Center for Chemical and Biological Sciences, University of Karachi, Karachi, 75270 Pakistan; 5 College of Arts and Sciences, American University of Sharjah, University City, Sharjah, United Arab Emirates

**Keywords:** Hesperidin, Silver nanoparticles, *E. coli* K1, Gene expression

## Abstract

**Backgrounds:**

*Escherichia coli* K1 causes neonatal meningitis. Transcriptome studies are indispensable to comprehend the pathology and biology of these bacteria. Recently, we showed that nanoparticles loaded with Hesperidin are potential novel antibacterial agents against *E. coli* K1. Here, bacteria were treated with and without Hesperidin conjugated with silver nanoparticles, and silver alone, and 50% minimum inhibitory concentration was determined. Differential gene expression analysis using RNA-seq, was performed using Degust software and a set of genes involved in cell stress response and metabolism were selected for the study.

**Results:**

50% minimum inhibitory concentration with silver-conjugated Hesperidin was achieved with 0.5 μg/ml of Hesperidin conjugated with silver nanoparticles at 1 h. Differential genetic analysis revealed the expression of 122 genes (≥ 2-log FC, *P*< 0.01) in both *E. coli* K1 treated with Hesperidin conjugated silver nanoparticles and *E. coli* K1 treated with silver alone, compared to untreated *E. coli* K1. Of note, the expression levels of cation efflux genes (*cusA* and *copA*) and translocation of ions, across the membrane genes (*rsxB*) were found to increase 2.6, 3.1, and 3.3- log FC, respectively. Significant regulation was observed for metabolic genes and several genes involved in the coordination of flagella.

**Conclusions:**

The antibacterial mechanism of nanoparticles maybe due to disruption of the cell membrane, oxidative stress, and metabolism in *E. coli* K1. Further studies will lead to a better understanding of the genetic mechanisms underlying treatment with nanoparticles and identification of much needed novel antimicrobial drug candidates.

**Supplementary Information:**

The online version contains supplementary material available at 10.1186/s12866-021-02097-2.

## Background

*Escherichia coli* is a commensal bacteria of the gastrointestinal tract of vertebrates, including humans [1]. It is also a Gram-negative bacterium that is involved in extraintestinal infections [2]. In particular, *E. coli* is the most frequent bacteria involved in preterm meningitis and the second most frequent cause of neonatal meningitis with a high mortality rate [3]. In spite of the widespread use of antibiotics in recent years, the incidence of bacterial meningitis is still in the range of 5 to 40%, and the neurological sequelae rate in survivors is up to 30% [4, 5]. Previous research has revealed that meningitis caused by *E. coli* K1 is contributed by several vital proteins, and several genetic islands, such as *sfa* (S fimbriae), *ksp* (K1 capsule), CNF-1 and *GimA*. Among these, *GimA* is one of the most unique and vital [6]. In recent years, resistance to available antibacterial agents by pathogenic bacteria has increased at an alarming rate and has become a serious problem [7]. The quest for alternative and novel antimicrobials is the demand in the present era. Approaches include the development of safe bio nanocomposites that have antibacterial activity and utilization of natural products.

Nanoparticles (NPs) are nano-dimensional materials, which act as a bridge between atomic and bulk materials. They have been shown to exhibit a variety of unique chemical, physical, biological and electronic properties [8]. The most biologically important of these NPs are of the noble metals such as silver, gold, and magnetic NPs due to their biocompatibility and other properties. Silver is a metal that has been widely used for NP synthesis since it is easily stabilized [9]. Metal NPs can be biologically synthesized by using bionanofactories natural sources such as cyanobacteria [10]. For example, the synergistic antibacterial activity enhancement of ciprofloxacin loaded metal NPs against MRSA was achieved by antibiotic combined with green synthesized CuFe_2_O_4_@Ag and/or NiFe_2_O_4_@Ag nanocomposites. In addition, both of nanocomposites efficiently reduced the expression of *norA* efflux pump gene that can reduce bacterial resistance to different antibacterial agents [11, 12]. Furthermore, natural phytochemical secondary metabolites such as alkaloids, flavonoids, carotenoids, and terpenoids are of growing interest for treatment. These compounds loaded on metal NPs have shown promising activities. Among flavonoids, the Hesperidin (HDN) is a major dietary flavanone which is abundantly found in many citrus fruits and exhibits a wide range of biological properties [13]. In the chemical skeleton of HDN, glucose is bonded to aglycone based structure (hesperetin) and rhamnose is bonded to this structure from glucose moiety [14]. Moreover, HDN has been recognized as a potent anti-inflammatory, anti-carcinogenic and antioxidant agent [15]. It exerts a higher inhibitory activity against Gram-positive bacteria than Gram-negative bacteria [16]. In addition, HDN can penetrate the blood–brain barrier and possesses neuroprotective actions [17].

Recent work in our laboratory depicted that silver nanoparticles (AgNPs) loaded with the flavonoid HDN exhibited potent bactericidal effects against *E. coli* K1, the causative agent of neonatal meningitis. In this study we analysed the differential gene expression (DGE) following the treatment of *E. coli* K1 with HDN conjugated AgNPs (AgNPs-HDN), in comparison to untreated *E. coli* K1, as well as those treated with unconjugated silver AgNPs. Such work will lay the foundations in furthering our comprehension of the genetic mechanisms of *E. coli* K1 in response to treatment with anti-bacterial drugs and to assist in the development of much required chemotherapies.

## Results

### Effect of compounds on the growth rate of *E. coli* K1

**Fig. 1:**
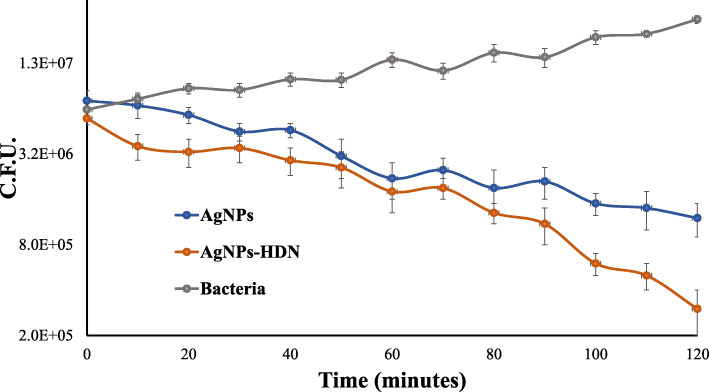
Growth curves of *E. coli* K1 grown in nutrient broth at 37 °C. Colony forming units of *E. coli* K1 in absence (grey line) or presence of 0.5 μg/mL HDN conjugated AgNPs (red line) or 0.5 μg/mL unconjugated AgNPs (blue line). Bacteria were counted at different time points. Each of the C.F.U. value represents the mean of duplicates

Growth curve analysis revealed that untreated *E. coli* K1 showed the highest growth rate at 37 °C (Fig. 1). The number of viable bacteria cultured with silver nanoconjugate samples gradually fell over time after 2 h of incubation. These data indicate that *E. coli* K1 was affected and diminished at 0.5 μg/ml of each AgNPs, AgNPs-HDN after 2 h incubation and displayed 50% killing of *E. coli* K1 after treatment of 1 h. Based on these observations, one-hour incubation of 0.5 μg/mL of NPs was deemed appropriate for RNA extraction.

### RNA gel electrophoresis analysis

The amount of small broken RNA, shown as thickness, for untreated *E. coli* K1 (columns 5,6) was slightly increased after exposure to AgNPs (3, 4 columns) and more visibly increased in both columns 1 and 2 after treatment with AgNPs-HDN (Fig. S1).

### Quantitative analysis of global gene expression

**Fig. 2:**
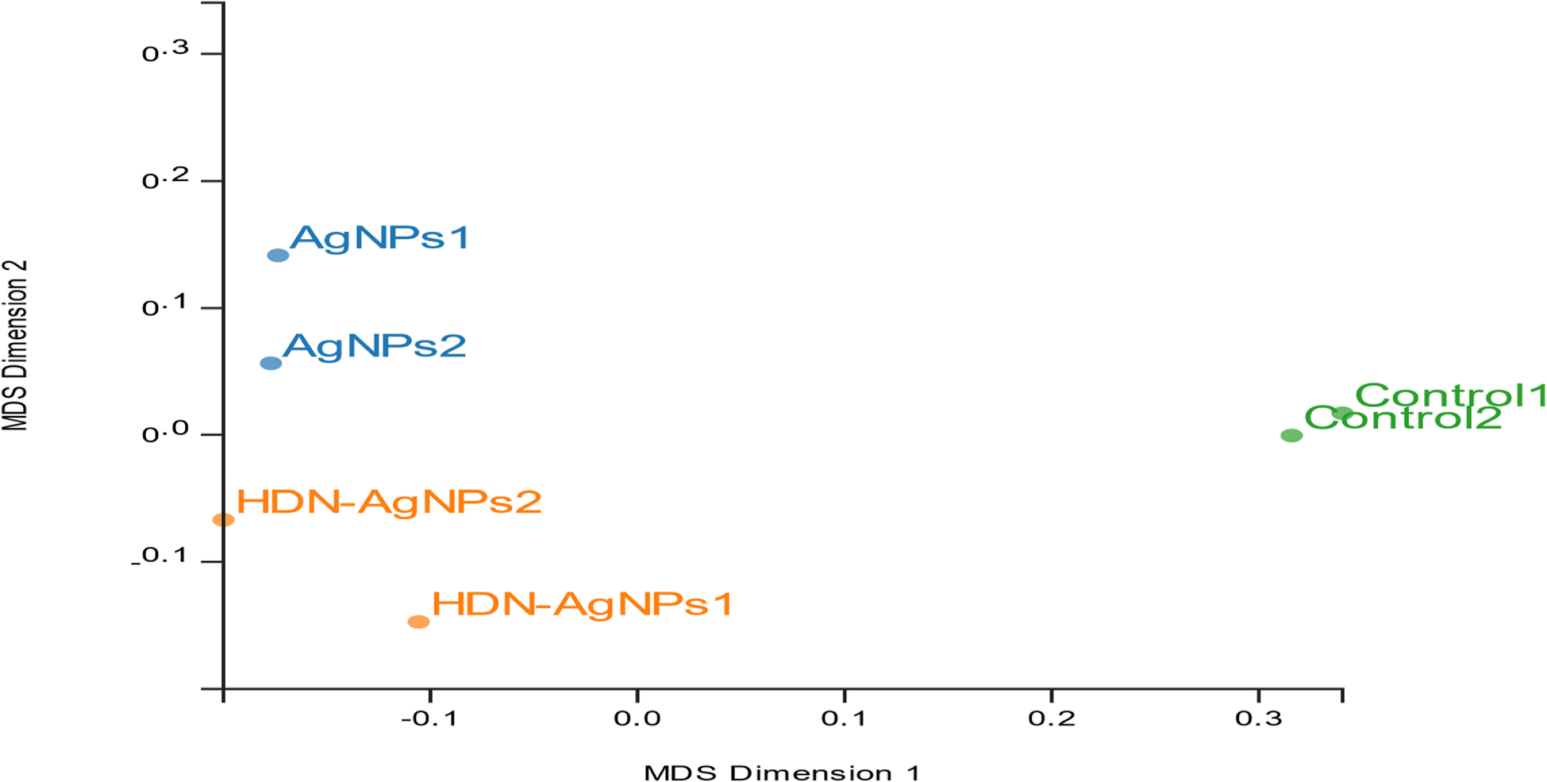
Multidimensional scaling plot. Distance between sample labels indicates relative similarity of samples

**Fig. 3:**
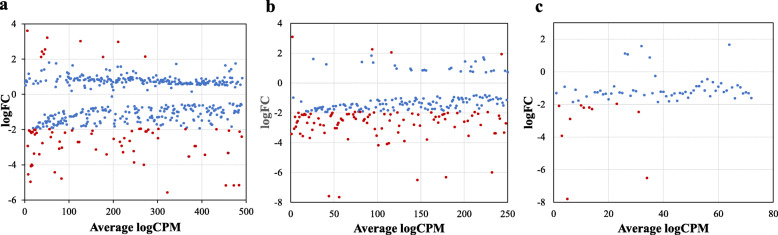
PlotSmear graph of the samples. Panel **a** Genes expressed in *E.coli* K1 treated with AgNPs vs *E.coli* K1 culture (control). Panel **b** Genes expressed in *E.coli* K1 treated with AgNPs- HDN vs *E.coli* K1 control. Panel **c** Genes expressed in *E.coli* K1 treated with AgNPs vs *E.coli* K1 treated with AgNPs- HDN

**Fig. 4:**
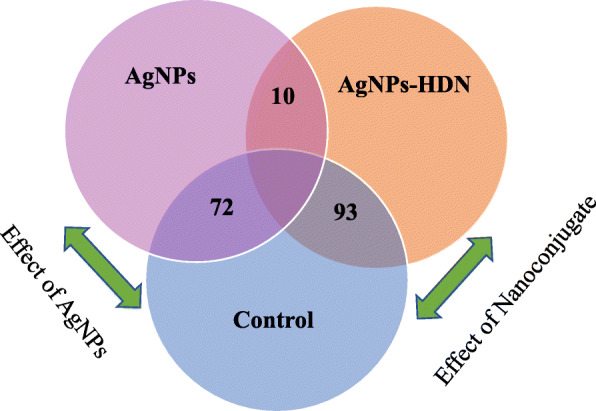
Venn diagram of the number of significant DEG among the different biological groups. Three comparison were made: AgNPs/*E.coli* K1alone; AgNPs- HDN/*E.coli* K1alone; AgNPs /AgNPs- HDN

The Multi-Dimensional Scaling (MDS) plot was been used to map the six samples on common/ 2-D space based on the similarity of their expression profiles. MDS showed that the biological replicates of the *E. coli* K1 through treatments and control were aggregated together (Fig. 2). The control samples showed more homogeneous than samples treated with AgNPs and HDN-AgNPs, suggesting a treatment effect. This indicates that the biological replicates are reliable and that multiple differentially expressed genes between treatments can be detected. Remarkably, there was more variability between the dissimilar experimental conditions than within every biological duplicate band. Figure 3 plots genes exhibiting significant expression changes through Critical Pathway Method (CPM) and parameters (log FC ≥2; FDR-adjusted *P*< 0.01) among the three pair-wise comparison groups. Overall, we identified 72 DGE (10 upregulated, 62 downregulated) between AgNPs and control groups (Fig. 3a); 93 DGE (4 upregulated, 89 downregulated) between AgNPs-HDN and control group (Fig. 3b), and 10 downregulated DGE between AgNPs and AgNPs-HDN groups (Fig. 3c). Ten genes were differentially expressed due to the effect of HDN as identified between AgNPs and HDN-AgNPs samples (Fig. 4). The top three downregulated DGE in nanoconjugate group were transcriptional regulator, multidrug transporter, and hypothetical protein. In addition, Azoreductase enzyme that have catalytic activity in response to oxidative stress. The top upregulated DGE in AgNPs was associated with multicopper oxidase, electron transporter and membrane protien.

**Fig. 5:**
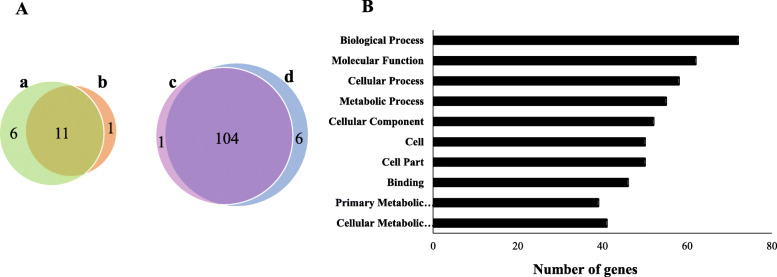
(A) Venn diagram for deferentially expressed contigs/genes (≥2-log FC) is depicted. a & b refer to upregulated genes in *E. coli* K1after treatment with AgNPs and AgNPs-HDN respectively. c & d refer to genes downregulated after treatment with AgNPs and AgNPs-HDN respectively. (B) Most enriched Gene Ontology terms for *E. coli* K1treated with AgNPs and *E. coli* K1treated with AgNPs- HDN, compared with bacteria alone

DGE analysis significantly revealed the presence of 122 genes that were upregulated and downregulated in both *E. coli* K1 treated with Hesperidin conjugated silver nanoparticles and *E. coli* K1 treated with silver alone, compared to untreated *E. coli* K1 (Fig. 5a). All of these 122 transcripts were further functionally classified into Gene Ontology (GO) categories, such as molecular functions, biological processes and cellular components to appreciate the putative function of DGE. Enriched GO terms totalled 10 terms. As shown in Fig. 5b, GO analysis identified a total of 5 terms related to cellular components, 4 terms for biological and metabolic processes, and 1term for molecular functions. Regarding cellular component ontology, most represented categories were translation, ions transportation, cell adhesion, and cellular metabolic process. Results showed that genes expression of treated *E. coli* K were up-regulated or down-regulated and indicated that play key roles in antibacterial effect. ***Gene expression profile of E. coli K1 in response to treatment.***

**Fig. 6:**
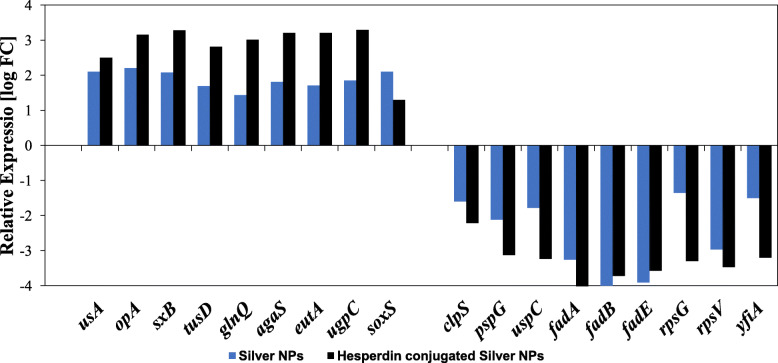
Representative gene expression profiles that are specific *E. coli* K1 treated with AgNPs and AgNPs-HDN (Abs-log FC≥2, adjusted *P* value < 0.01)

Among of DGE (122), eighteen genes that were differentially expressed by more than 2 log FC were selected. Nine genes were up-regulated and 9 genes were down regulated (Fig. 6). To understand the molecular basis of action against bacterial cells, genes were selected based on their functional annotations and sharing in various main functional classification according to GO and these genes are involved in metabolism, cell structures, and stress.

In response to AgNPs, the expression of *cusA* (encoding cation efflux system protein), *copA* (encoding copper-exporting P-type ATPase), and *rsxB* (encoding ion-translocating oxidoreductase complex subunit B) were upregulated 2.1, 2.2 and 2.0-log FC respectively. In addition, the same genes were also expressed higher when exposed to AgNPs-HDN (Fig. 6). Other up-regulated genes comprised of: *tusD* encoding subunit D of sulfurtransferase, *glnQ* encoding glutamine transporter ATP-binding protein. In addition, *agaS* codes for putative tagatose-6-phosphate ketose/aldose isomerise, *eutA* codes ethanolamine utilization protein, *ugpC* codes for sn-glycerol-3-phosphate import ATP-binding protein, and *soxS* (transcriptional regulator) had significant increase in the upregulated gene expression of AgNPs and AgNPs-HDN. Likewise, our analysis revealed that several genes were downregulated in *E. coli* K1 treated with HDN conjugated AgNPs, and AgNPs alone compared to untreated *E. coli* K1 (Fig. 6), Many of the downregulated genes showed significant decrease upon treatment. These genes include: *clpS* which is ATP-dependent protease protein, *pspG* (phage shock protein), *uspC* encode fimbria adhesion protein, fatty acid metabolism protein *fad A*,*B*,*E*, *rpsG* codes for ribosomal protein, *rpsV* codes for Stationary-phase-induced ribosome, the ribosome-associated inhibitor *yfiA*.

## Discussion

Neonatal bacterial meningitis is a destructive infection. Even though the incidence and mortality have dropped since the last few decades, morbidity among survivors is high*.* Nonetheless*, E. coli* K1 causes between 70 and 80% of cases of neonatal bacterial meningitis in developed countries [18]. To this end, nanotechnology has shown promise in the treatment of bacterial infections [19]. Our previous work has revealed that when *E. coli* K1 was treated with AgNPs-HDN, significant bactericidal effects were observed, along with reduced bacterial-mediated host cells cytotoxicity. Moreover, when tested against human cells, these NPs showed minimal cytotoxicity. Thus, these novel nanoparticle formulations could be utilised as therapeutic agents against infections caused by multi-drug resistant bacteria [15]. The virulence of *E. coli* K1 is related to the ability of the K1 capsule to inhibit phagocytosis and to resist antibody-independent serum bactericidal activity. In addition, K1 strains are able to cross the blood–brain barrier via their ability of adhering to host tissues by specific fimbrial proteins, for example one of the gene encoding the adhesin of these fimbriae is *fimH* [20]. However, the genetic mechanisms by which these nanoparticles interact with *E. coli* K1 remains poorly understood.

In this study we accomplished transcriptome investigation to analyse DGE in *E. coli* K1, following treatment with AgNPs loaded with the citrus fruit flavonoid HDN. The gene expression analysis of 122 genes (≥ 2-log FC) in both *E. coli* K1 treated with AgNPs- HDN and *E. coli* K1 treated with AgNPs were differentially expressed as compared to untreated *E. coli* K1, (*P*< 0.01) (Fig. 6). Previous work has suggested that the primary cause of nanoparticle antibacterial function might be from the induction of reactive oxygen species (ROS), including hydrogen peroxide that can penetrate into bacterial cells [20]. These ROS exhibit high reactivity to all components including lipid, protein, and nucleic acids. In order to understand the effects of NPs on cells and organisms, several mechanisms have been proposed including oxidative stress induction, cell membrane and components disruption, and cell function interference [21]. For instance, as response to the presence of sub-inhibitory concentration of Ag@Glu/Tsc NPs, the expression of biofilm formation genes icaA and icaD involved with the synthesis of intercellular adhesion molecules reduced by 66.7 and 60.3%, respectively [22]. In addition, previous work depicting global transcriptome analyses revealed that flavonoids (phloretin) repressed toxin genes (*hlyE* and *stx2*), autoinducer-2 importer genes (*lsrACDBF*) and structural subunit of the curli fimbriae genes (*csgA* and *csgB*) in *E. coli* O157:H7 [23].

Bacteria have a great capacity for adjusting their metabolism in response to environmental changes by linking extracellular stimuli to the regulation of genes by transcription factors and in certain cases, transcription regulators control genes and operons that belong to different metabolic pathways [24]. Moreover, bacteria adopt mechanisms that quickly regulate the synthesis of defensive proteins in response to stress [25]. From our experiments in *E. coli* K1, the antibacterial mechanism of both nanoparticles maybe through increased levels of oxidative stress, which affect mainly on the metabolism of bacterial cells [26]. But we would further expect to see other stress genes associated with oxidative stress.

Interestingly, our study revealed that the *cusA* and *copA* genes were upregulated when *E. coli* K1 was treated with AgNPs-HDN. Studies have shown that in Gram-negative bacteria, these genes play an important role in transport of copper across the cytoplasmic and outer membrane [27]. Previous studies have reported that concentration of Ag+ activated expression of a heavy metal efflux pump which is encoded by *cusCFBA* operon, formed from central *CusA*, that spans the cell envelope, and a small periplasmic metal binding protein, *CusF* that may sequester the Ag+/Cu+ or chaperone the ions to the efflux complex. Additionally some studies revealed that *CueR* interacts with Ag+/Cu+ and positively regulates the expression of *copA*, encoding a copper oxidase, producing a Cu+−translocating P-type ATPase, exports Cu+ from the cytoplasm to the periplasm and may also be involved in silver export [28, 26]. These mechanisms could be potential targets in developing novel therapies.

Despite of presence of slight significant upregulated expression of *soxS* (2.1, 1.3-log FC for AgNPs, AgNPs-HDN respectively) which is transcriptional activator of the superoxide stress response genes, results from our study depicted the absence of significant expression of genes such as *katG*, *katE*, *sodA*, *sodB*. To illustrate, the observed 3.2-log fold upregulation of *rsxB* in *E. coli* K1 treated with AgNPs- HDN diminish oxidation of SoxRS regulon and maintain reduction state. These genes were probably be due to insufficient SoxR oxidation which results from up regulation of *rsxB*. Furthermore, another SoxS regulated gene *gltA* (encoding enzyme have catalytic activity in krebs cycle involved in the respiratory pathway) [24]. Our results showed that *gltA* is weakly downregulated and that corresponds with the low oxidation level of SoxS.

Results from our study revealed that the *tusD* gene was upregulated in both *E. coli* K1 treatments. TusBCD and the protein TusE belong to the sulphur relay system involved in thiouridine biosynthesis [28]. Sulphur is essential to form protein by complex processes involving the successive transfer of sulphur between multiple proteins, The L-cysteine desulfurase (IscS) mobilizes sulphur from L-cysteine for the synthesis of several biomolecules and the mobilization process is mediated by different interaction partners such as TusA which interacts at specific site of IscS and stimulates its activity 3-log fold. The sulphur on TusA is then transferred to TusD in the TusBCD complex and transfers it to tusE. Finally, TusE interacts with tRNA complex for thiomodification [29]. How, this pathway could be exploited in future studies needs to be determined, and this could be a potential pathway in developing novel drug targets.

Furthermore, our data indicated 1.6, 2.2-log fold expression downregulation for *clpS* in both *E. coli* K1 treated with silver alone and AgNPs -HDN respectively. These are part of five known ATP-dependent proteases in *E. coli* (Lon, ClpAP, ClpXP, HslUV, and the membrane-associated FtsH) that catalyze the removal of both misfolded and properly folded proteins in cellular protein quality control pathways [30]. In the bacterial cytosol, ATP-dependent protein degradation is performed by different chaperone- protease pairs, including the heat shock response genes *ClpAP*. Degradation function of this complex is mediated by ClpS which directly influences the ClpAP machine by binding and modifying ClpA, potentially redirecting degradation of ClpAP toward aggregated or unfolded proteins while at the same time inhibiting degradation of non-aggregated substrates [31]. This is of interest and these pathways ought to be further investigated.

Previous studies have shown that the phage shock protein (Psp) stress response system is responsible for repairing damage to the inner membrane of the cell and to maintenance of the proton-motive force (pmf) across the inner membrane, the *pspABCDE* operon together with *pspF* and *pspG* form the *Psp* regulon [32]. Furthermore, another member of stress response system- *usp* gene is functionally distinct from its family members which is not involved in stress resistance but is essential for cellular motility. Fimbria-mediated adhesion is increased by UspC protein [33]. Studies have shown that the deletion of *uspC* gene results in sensitivity to DNA damaging agents [33]. Also, Ag ions can bind to nucleic acid but DNA is typically localised to the core of the cell even its surrounded by high concentrations of proteins which will be attacked first [34]. Accordingly, at the inhibitory dose, our outcomes showed that there is evidence for a genotoxic response such as upregulation of *recR* gene one-log fold with silver alone and 1.5-log fold after applying HDN conjugated AgNPs, and that may play a role in DNA repair [35]. In addition, our research displayed that *glnQ* gene was up regulated 3- log fold after exposure to AgNPs-HDN, the gene encodes a protein, which is part of the binding-protein-dependent transport system for glutamine, is present in the inner membrane, probably responsible for energy coupling to the transport system [36].

Bacteria are known to possess flagella which are complex molecular machines critical for cell motility and chemotaxis, and the expression of flagellar genes is highly regulated [37]. More than 50 genes in *E. coli* are thought to participate in the formation and manoeuvring of the flagellum [38]. Synchronised expression of flagellar genes is coordinated by regulatory proteins, with flhDC and fliA (*fliA* encodes RNA polymerase sigma factor for flagella) as master transcriptional activators [39]. Our DGE analysis revealed that the *fliA* gene was downregulated with less than 2-log FC in *E.coli* K1 when treated with AgNPs-HDN and also when treated with silver alone. Moreover, *fliF* was slightly upregulated suggesting that the flagellar M ring protein may be actively involved in energy transduction. This is corroborated by a similar previous study where the downregulation of *fliA* and *fliF* genes were observed when *E. coli* were exposed to AgNPs by 3.3 and 2.7-log fold respectively [40]. Moreover, the genes (*fadA*, *fadB*, *fadE*) were downregulated more than 3-log folds only after exposure to AgNPs and AgNPs-HDN. This is possibly due to localized membrane damage leading to an increase in the expression of these genes. Other ribosomal metabolic genes that were downregulated in our study include the *rpsG* gene, which has role in expression regulation of the str operon members. In addition the *rpsV* gene that is involved in translation [34], and also the *yfiA* gene that is responsible for stabilization and regulation of 70S ribosome translation during the stationary phase and *yfiA* gene consider as one of the most strongly H_2_O_2_- induced genes in wild type strain of *E. coli* [41].

Further analysis of nanoparticle *E. coli* K1 gene expression showed that the transcription levels of two metabolic and one cellular process gene: *agaS*, *eutA*, and *ugpC* were significantly increased, by up to 3-log fold. It is indeed not surprising to find that several metabolic genes were up-regulated and these include the *agaS* gene (required for galactosamine utilization) [42], *eutA* gene that codes the protein involved in the ethanolamine degradation pathway [43]. A pathway analysis will provide more insight. Lastly, the transport/binding gene *ugpC*, responsible for energy coupling to the transport system. Furthermore, another 2 biosynthesis of amino acid/ aspartate family genes (*thrA* and *aspA*) were expressed at approximately 1.5- to 1.8-times lower levels. These genes have catalytic activity [44].

## Conclusions

Here we have demonstrated for the first time, DGE in *E. coli* K1 following treatment with HDN conjugated AgNPs, silver, and untreated *E. coli* K1*.* The gene expression analysis revealed 122 genes (≥ 2-log FC) in both *E. coli* K1 treated with HDN conjugated AgNPs and *E. coli* K1 treated with silver alone were differentially expressed as compared to untreated *E. coli* K1, (*P*< 0.01). In summary, silver nanoparticles at low concentrations induced (up to 6-fold) expression of different type of *E.coli* K1genes. The common mechanism of NPs inactivation of bacteria involves the direct interaction between NPs and cell surfaces, which affects the permeability of membranes where nanoparticles enter and induce oxidative stress, subsequently resulting in the inhibition of cell growth and eventually in cell death [25]. Based on our data, the differential AgNPs and AgNPs-HDN response support a growing body of evidence for a nanoparticle-specific silver ion dependent toxicity mechanism. There was clear evidence that Ag causes redox stress, as well as response to proteins were observed, reflecting the metabolism action of silver on protein structure and function. Accordingly, we consider that this process is the primary mechanism in Ag toxicity against *E. coli* K1. In order to fully understand the antibacterial mechanism of AgNPs and their conjugates, it will be necessary to conduct future studies. These genes may be effective targets in the expansion of chemotherapeutic interventions. Subsequent studies will determine the precise molecular pathways.

## Methods

### Chemicals and characterization of HDN gum stabilized nanoparticles

The regents and chemicals used for the assays in this study comprise of HDN and Silver nitrate (Sigma Aldrich, United Kingdom), unless stated otherwise. Deionized water was used for all the formulations. Both the synthesis of HDN gum stabilized nanoparticles and characterization of the nanoparticles size, Zeta potential, Transmission electron microscope (TEM), Fourier transform infrared spectroscopy (FTIR) and polydispersity index (PDI), were conducted previously, as described [15].

### Bacterial growth curve analysis

Bacteria utilised in this study were as previously described [15]. Namely, neuropathogenic *Escherichia coli* K1 (a cerebrospinal fluid isolate from a meningitis patient; O18:K1:H7) (MTCC 710859) was cultivated. Several colonies of *E. coli* K1 were suspended in sterile nutrient broth. Following 10 h the optical density (OD) was adjusted to 595 nm to obtain a bacterial concentration of approximately 10^8^ colony-forming units (CFU)/ml [45]. Ten microliters of *E. coli* K1 suspension were added to Phosphate Buffer Saline (PBS) containing 0.5 μg/ml AgNPs, or 0.5 μg/ml AgNPs-HDN. PBS alone was utilised as a control. Cultures were then treated with the aforementioned, in duplicates at various time intervals (0, 10, 20, 30, 40, 50, 60,70, 80, 90, 100, 110 and 120 mins). Following treatment, 10 μL of each aliquot were serially diluted and plated on nutrient agar in duplicates, followed by incubation at 37 °C for 16 h before bacterial colonies were enumerated.

### *E.coli* K1 RNA isolation

To extract total cellular RNA, the conventional bacterial RNA extraction method was performed. The total RNA was extracted from untreated *E.coli* K1, *E.coli* K1 treated with AgNPs-HDN and treated with silver alone in duplicates. The concentrations and times used herein were determined from the results obtained in the growth curve analysis mentioned above. Cells were harvested by centrifugation at 4000 g for 10 min at 4 °C, and all subsequent steps were performed on ice. Briefly, 200 μL of solution (95% ethanol+ 5% phenol) was added to 10^6^ C.F.U. *E.coli* K1 pellets were centrifuged at 10000 rpm for 10 mins and the supernatant was discarded completely. Next 1.2 ml TRIzol™ was added quickly, and the pellet was resuspended carefully, and incubated for 5 mins to lyse the cells. Then 200 μl of chloroform was added and mixed by inversion of the tube and then transferred again and incubated for 5 mins and centrifuged at 13000 rpm for 15 mins. Finally, the top clear layer was transferred into a new tube and 500 μL isopropanol was added to precipitate RNA and incubated for 15 mins in -20 °C. Next the tubes were centrifuged at 13000 rpm for 10 mins and the supernatant was discarded. This was washed twice with 1000 μl 75% pre-chilled ethanol followed by centrifugation at 13000 rpm for 5 mins. The supernatant was discarded after each wash and the pellet was dried in air (with the tube opened in the Biosafety hood) for a few minutes until a translucent jelly appearance was observed. After that, 30 μL RNase free water was added and re-suspended by gentle flicking and incubated for 5–10 min on a heat block with water at 50-60 °C [46]. Extracted RNA was treated with DNaseI (Fermentas, Hanover, MD) to remove genomic DNA contamination. RNA concentration and quality were assessed using the Nanodrop UV-Vis Spectrophotometer. Pure RNA samples comprise of a ratio between 1.8 and 2.1 when measured at the spectrophotometric relative absorbance ratio at (260 nm/280 nm).

### RNA gel electrophoresis

The RNA of the 3 samples in duplicates (*E.coli* K1 treated with 0.5 μg/ml AgNPs-HDN, *E.coli* K1 treated with 0.5 μg/ml AgNPs, and untreated bacteria) were run on a gel. Briefly, the gel was prepared using 1–1.2% (1X) TEA buffer, heated with mixing until dissolved and 1 μl of gel dye was added while mixing. The gel was poured into a gel tray and allowed to solidify. The tray was transferred into a gel tank and filled with buffer. Next 1 μl of tracking dye was mixed with 5 μl sample. The samples were heated until 95-120 °C for 1 min and placed on ice prior to loading. A 1 kb DNA ladder (5 μl) was pipetted into the first well and the other samples were pipetted in other wells. The gel was run at 100 V for 40 mins and imaged to visualise bands of bacterial RNA 1800 23S and 1200 16S bands. The RNA integrity and concentrations were also checked using Qubit 2.0 fluorometer and the RIN (RNA integrity number) was assessed using Agilent TapeStation 2200 (Table 1).

**Table 1:** The criteria of bacterial RNA samples. A0: control, A and B: *E. coli* K1 treated with AgNPs-HDN, C and D: *E. coli* K1 treated with AgNPs, E and F: untreated *E. coli* K1

Well	RIN	23S/16S (Area)	Conc. (ng/μl)
**A0**	–	–	84.9
**A**	7.2	1.0	239
**B**	7.4	1.1	236
**C**	7.5	1.2	217
**D**	7.3	1.1	229
**E**	7.6	1.3	177
**F**	7.8	1.4	109

### cDNA library preparation and sequencing

Ribosomal RNA depletion was performed using the MICROB *Express* Bacterial mRNA Enrichment kit (Thermo Fisher Scientific, Massachusetts, USA) following the manufacturer’s protocol. The enriched mRNA was subjected to cDNA library preparation using the NEBNext Ultra RNA Library Prep Kit for Illumina (New England Biolabs, Massachusetts, USA). The library was then quantified using Qubit 2.0 fluorometer (Invitrogen, California, USA) and the size distribution was checked using TapeStation 2200 (Agilent Technologies,California, USA). All libraries were pooled equimolar and 15pM was loaded into the MiSeq cartridge. Sequencing was performed on the MiSeq system using a 2 × 75 bp run configuration.

### Bioinformatics analyses

The reference genome utilised were the *Escherichia coli* RS218 (O18:H7:K1) and their NCBI assembly accessions were CP007149 and CP007150 for plasmid sequence [47]. An index file of this genome was used for mapping using Bowtie2 v2.3.5 [48]. Genome annotation was done using Prokka, supplemented with *Escherichia coli* K12 annotation using accession GCF_000005845.2 [49]. Illumina adapter sequences and low-quality bases were trimmed off using Trimmomatics v0.39 using the parameters ILLUMINACLIP:adapters.fa:2:30:10:1:true, SLIDINGWINDOW:5:20, LEADING:20, TRAILING:20 [50]. The trimmed, quality-filtered reads were mapped to the *E.coli* RS218 index file using Bowtie2 v2.3.5 and samtools v1.9 [48]. The counting of the alignments on annotated genes was done using htseq-count v0.11.2 using parameters mode = union, order = pos and minaqual = 0 [51]. For differential expression analysis, raw read counts were imported and analyzed using the Degust software and the genes were identified using EdgeR. Gene regulation was subject to confidence testing and filtered using Abs-log FC≥2) greater than 4-FC cut-off and 0.01 as false discovery rate (FDR) to generate lists of up-regulated and down-regulated genes. To cluster the samples based on the similarity of gene expression profiles multi-dimensional scaling (MDS) was applied. Finally, DGE were then subjected to Gene Ontology Enrichment Analysis using ShinyGO v0.61 software utilizing default parameters. GO analysis using the lists of differentially expressed genes revealed how they are mutually involved in a number of biological processes. The submission to NCBI SRA has been completed and the BioProject ID is PRJNA629415.

## Supplementary Information

**Additional file 1 Fig S1**. Agarose gel electrophoresis showing 16S and 23S rRNA fragments for the six isolates. Lane M: 1 kb DNA ladder; Lanes number 1 and 2: *E. coli* K1treated with AgNPs-HDN; Lanes 3 and 4: *E. coli* K1 treated with AgNPs; Lanes 5 and 6: Untreated *E. coli* K1.

## Data Availability

The datasets generated during the current study are available in the NCBI SRA repository, BioProject ID is PRJNA629415.

## Abbreviations

*E. coli*: *Escherichia coli*
NPs: Nanoparticles
HDN: Hesperidin
AgNPs: Silver nanoparticles
DGE: Different gene expression
AgNP-HDN: Hesperidin conjugated silver nanoparticles
MDS: Multi-Dimensional Scaling
GO: Gene Ontology
RNA: Ribonucleic acid
rRNA: Ribosomal ribonucleic acid
mRNA: Messenger ribonucleic acid
tRNA: Transfer ribonucleic acid
DNA: Deoxyribonucleic acid
ATP: Adenosine triphosphate
PDI: Polydispersity index
OD: Optical density
CFU: Colony forming units
PBS: Phosphate Buffer Saline
RIN: RNA Integrity number
FDR: False discovery rate
NCBI: National Center for Biotechnology Information
SRA: Sequence Read Archive
