# Supplementary figures and images for: Transcriptome analysis of Escherichia coli K1 after therapy with hesperidin conjugated with silver nanoparticles
Source: BMC Microbiol. 2021 Feb 17;21:51. doi: 10.1186/s12866-021-02097-2 (PMC7890611; doi:10.1186/s12866-021-02097-2)

## Slide 1
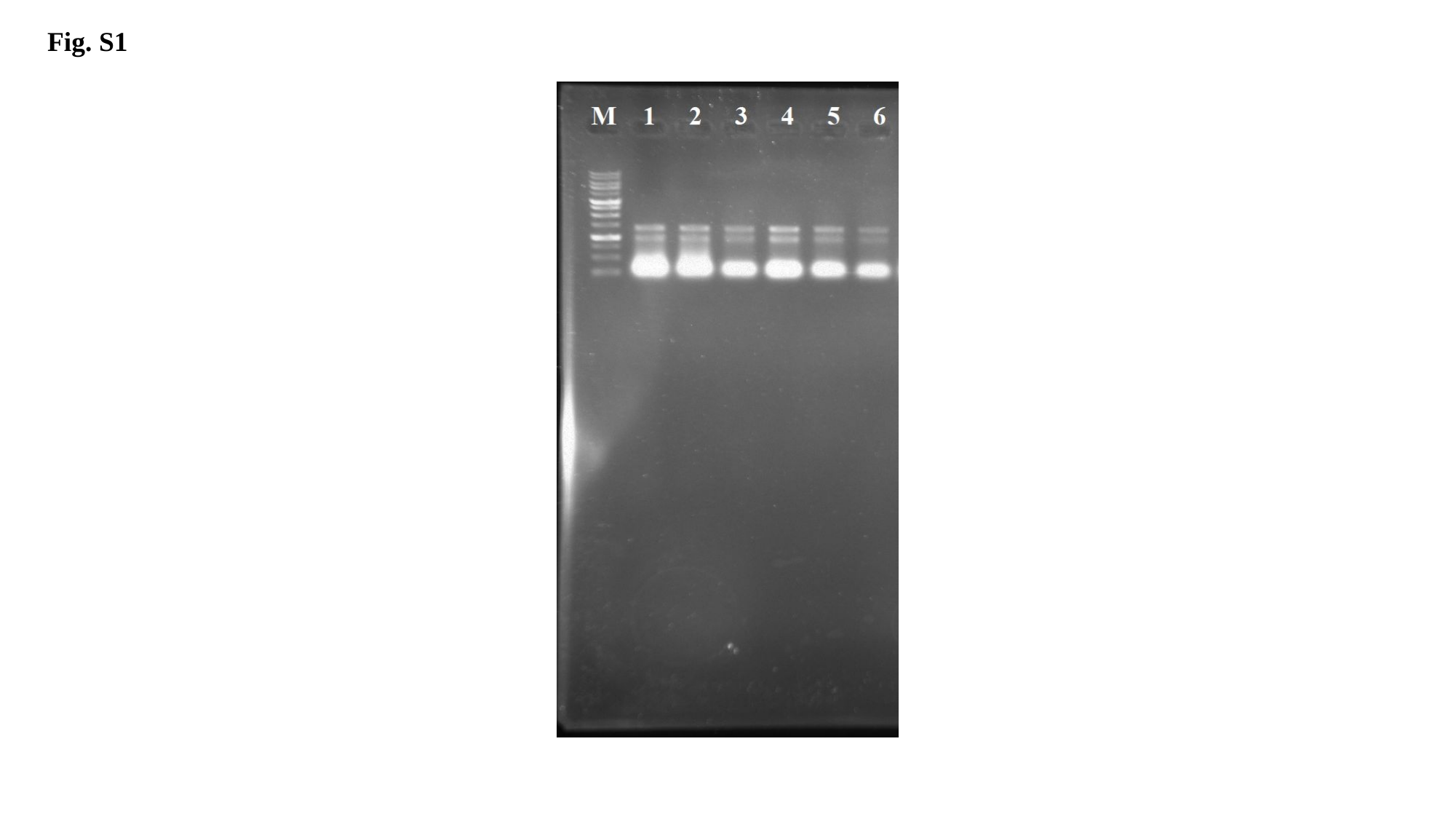

Fig. S1

Supplement: Supplementary file 1 — Additional file 1 Fig S1. Agarose gel electrophoresis showing 16S and 23S rRNA fragments for the six isolates. Lane M: 1 kb DNA ladder; Lanes number 1 and 2: E. coli K1treated with AgNPs-HDN; Lanes 3 and 4: E. coli K1 treated with AgNPs; Lanes 5 and 6: Untreated E. coli K1. [file 12866_2021_2097_MOESM1_ESM.pptx]
